# Telehealth-Supported Decision-making Psychiatric Care for Suicidal Ideation: Longitudinal Observational Study

**DOI:** 10.2196/37746

**Published:** 2022-09-30

**Authors:** Erin O'Callaghan, Nicole Mahrer, Heather G Belanger, Scott Sullivan, Christine Lee, Carina T Gupta, Mirène Winsberg

**Affiliations:** 1 Brightside Health Inc Oakland, CA United States; 2 Psychology Department University of La Verne La Verne, CA United States; 3 Department of Psychiatry and Behavioral Neurosciences University of South Florida Tampa, FL United States

**Keywords:** telemedicine, telehealth, psychiatry, mental health, suicidal ideation, depression, anxiety, suicide, depressive disorder, digital health, eHealth, psychiatric medication, demographic, psychiatric care, decision-making, decision support

## Abstract

**Background:**

Suicide is a leading cause of death in the United States, and suicidal ideation (SI) is a significant precursor and risk factor for suicide.

**Objective:**

This study aimed to examine the impact of a telepsychiatric care platform on changes in SI over time and remission, as well as to investigate the relationship between various demographic and medical factors on SI and SI remission.

**Methods:**

Participants included 8581 US-based adults (8366 in the treatment group and 215 in the control group) seeking treatment for depression, anxiety, or both. The treatment group included patients who had completed at least 12 weeks of treatment and had received a prescription for at least one psychiatric medication during the study period. Providers prescribed psychiatric medications for each patient during their first session and received regular data on participants. They also received decision support at treatment onset via the digital platform, which leveraged an empirically derived proprietary precision-prescribing algorithm to give providers real-time care guidelines. Participants in the control group consisted of individuals who completed the initial enrollment data and completed surveys at baseline and 12 weeks but did not receive care.

**Results:**

Greater feelings of hopelessness, anhedonia, and feeling bad about oneself were most significantly correlated (*r*=0.24-0.37) with SI at baseline. Sleep issues and feeling tired or having low energy, although significant, had lower correlations with SI (*r*=0.13-0.14). In terms of demographic variables, advancing age and education were associated with less SI at baseline (*r*=−0.16) and 12 weeks (*r*=−0.10) but less improvement over time (*r*=−0.12 and −0.11, respectively). Although not different at baseline, the SI expression was evident in 34.4% (74/215) of the participants in the control group and 12.32% (1031/8366) of the participants in the treatment group at 12 weeks. Although the participants in the treatment group improved over time regardless of various demographic variables, participants in the control group with less education worsened over time, after controlling for age and depression severity. A model incorporating the treatment group, age, sex, and 8-item Patient Health Questionnaire scores was 77% accurate in its classification of complete remission. Those in the treatment group were 4.3 times more likely (odds ratio 4.31, 95% CI 2.88-6.44) to have complete SI remission than those in the control group. Female participants and those with advanced education beyond high school were approximately 1.4 times more likely (odds ratio 1.38, 95% CI 1.18-1.62) to remit than their counterparts.

**Conclusions:**

The results highlight the efficacy of an antidepressant intervention in reducing SI, in this case administered via a telehealth platform and with decision support, as well as the importance of considering covariates, or subpopulations, when considering SI. Further research and refinement, ideally via randomized controlled trials, are needed.

## Introduction

### Background

Suicide is a leading cause of death in the United States, claiming the lives of >47,000 people in 2019 [1]. Furthermore, the prevalence of suicidal ideation (SI) is high, with 12 million adults endorsing suicidal thoughts in 2019 [1].

Amid the current global COVID-19 pandemic, concerns arose about increases in SI and suicide, with a study suggesting a particularly heightened risk at the intersection of patient vulnerability, risk, resources, and mental health status [2]. Although suicide rates remained largely unchanged or declined in the early months of the pandemic compared with the expected levels based on the prepandemic period [3], rates among adolescents [4] and young adults have increased in the aftermath [5]. A recent survey revealed significantly elevated rates of SI in those aged 18 to 24 years, minority groups, unpaid caregivers, and essential workers [6]. Overall, these trends emphasize a critical need to better understand the predictive risks of suicide and effective mediation.

Although 90% of those who commit suicide have a psychiatric diagnosis [7], predicting who will attempt suicide is difficult. SI, defined as “thinking about, considering, or planning suicide” [8], is predictive of suicide attempts and completion [9,10]. In addition, SI is a better predictor of lifetime risk for suicide than imminent risk [11]. It is estimated that among those endorsing SI, there is a 29% conditional probability of making a suicide attempt [12]. In a large retrospective study, those with nearly daily SI were 5 to 8 times more likely to attempt suicide and 3 to 11 times more likely to die by suicide within 30 days [13]. The effects of treatment with common antidepressants on SI are mixed [14-16], with the most recent review suggesting that they are associated with a higher risk of suicide [17].

Several studies have demonstrated that depression is the most common psychiatric disorder among people who die by suicide, with an estimated 50% to 75% diagnostic prevalence in suicide cases [18,19]. In addition, anxiety disorders, particularly general anxiety disorder (GAD), may be independently associated with SI and suicide attempts [20,21]. Panic disorders and attacks are associated with an increased risk of SI and suicide attempts [22]. Rates of both anxiety and depressive disorders have increased considerably in the United States in recent years, with a notable spike between April 2020 and June 2020 compared with the same period in the previous year [23], which has been suggested as a potential result of the impact of the COVID-19 pandemic on global mental health. In addition, drug and alcohol abuse has increased during the pandemic [24], both of which are associated with an increased risk for SI and suicide [25,26].

In addition to the mental health conditions associated with an increased risk of suicide, certain physical health conditions such as chronic pain and chronic medical conditions have also been shown to be associated with increased SI and suicide attempts [27-31]. Approximately 20% of the individuals with chronic pain endorse SI [28], while 48% of the patients with fibromyalgia endorse SI [32]. Those with >1 chronic medical conditions have similarly elevated rates of SI, with 35% of those with ≥2 conditions endorsing lifetime SI [30]. After controlling for major depression and associated symptoms, as well as various demographic factors, the presence of a chronic medical condition was associated with a 1.3-times increase in the likelihood of SI [30].

Various demographic variables have been investigated as potential risk factors for SI and suicide. In general, factors such as sex (male), ethnicity (White, American Indian, or Alaska Native individuals), education level (high school or less), and economic factors (unemployment) are associated with higher rates of suicide [33-35]. Although women are more likely to have SI, men more often die by suicide [34]. Similarly, despite a low prevalence of SI in White men aged >75 years, they have one of the highest rates of fatality by suicide [11].

It has been relatively well established that suicide has a strong association with psychiatric disorders, especially major depressive disorder, and that pharmacological and nonpharmacological methods are often indicated for patients expressing SI as part of depressive symptomology. The course of treatment may commonly include prescribing antidepressants, such as selective serotonin reuptake inhibitors, serotonin-norepinephrine reuptake inhibitors, more modern antidepressants such as bupropion, older tricyclic antidepressants, and monoamine oxidase inhibitor antidepressants. Although antidepressants are a common treatment route, overall, there are conflicting findings regarding whether they reduce SI or suicide, or both [17,36-40]. The literature reveals a mixed and inconsistent understanding of their therapeutic effects with respect to SI and suicide. Furthermore, some studies have suggested that antidepressants may worsen suicidality in children and young adults [41,42], although this has been disputed [43,44].

### Objective

Given the limited and inconsistent understanding of the effects of psychotropic treatment on SI, this study seeks to add to the literature by investigating the impact of psychiatric care, delivered via a telehealth platform, on SI. The objective of this study was, therefore, to examine the impact of this psychiatric care platform on SI, change in SI over time, and remission, as well as to investigate the relationship between various demographic and medical factors on SI and SI remission.

## Methods

### Participants

Participant data used in this study were obtained from a national mental health telehealth company (ie, Brightside) and consisted of 8581 US-based patients receiving psychiatric care for depression or anxiety, or both between October 2018 and April 2021 (treatment, n=8366; control, n=215). Participants were eligible if they (1) completed surveys at baseline and at 12 weeks; (2) denied any history of psychosis, schizophrenia, or bipolar I disorder; and (3) denied any history of chronic liver or kidney disease. Participants in the control group met the same criteria and signed up initially for Brightside but did not receive care. Brightside uses a free self-care product that sends emails requesting the completion of survey data over a period of 14 weeks even with no sign-up. The control group therefore consisted of individuals who completed the initial enrollment data and completed surveys at baseline and at 12 weeks. The treatment group included individuals who engaged in treatment with Brightside for at least 12 weeks. The demographic and clinical characteristics of the 2 groups are shown in Table 1. As evident in Table 1, the treatment group had significantly greater depression severity at baseline. The control group was more likely to have a high school education or less and was more likely to be unemployed. There were no significant differences on other baseline demographic or clinical characteristics, including initial SI and suicide attempts.

**Table 1:** Demographic and clinical characteristics of the sample by group.

Characteristic			Treatment (n=8366)		Control (n=215)		*P* value
Suicidal ideation^a^ (baseline), mean (SD)			0.77 (0.98)		0.80 (1.04)		.63
Patient Health Questionnaire-8 score, mean (SD)			16.92 (4.38)		16.15 (5.06)		.01
Generalized Anxiety Disorder-7 score, mean (SD)			14.81 (4.52)		14.69 (4.82)		.69
Age (years), mean (SD)			32.02 (8.70)		31.97 (10.42)		.94
Sex (female), n (%)			5928 (70.86)		122 (74.39)		.34
**Racial minority^b^, n (%)**							
	Not White	1727 (20.64)		38 (25.33)		.16	
	White	6639 (79.36)		112 (74.67)		.16	
	Black or African American	296 (3.54)		9 (6)		.16	
	Asian	286 (3.42)		4 (2.67)		.16	
	Hispanic	671 (8.02)		16 (10.67)		.16	
	Other	474 (5.67)		9 (6)		.16	
Education (beyond high school), n (%)			5727 (68)		78 (52)		<.001
**Employment status, n (%)**							
	Full-time	5738 (68.59)		135 (63.08)		.02	
	Part-time	975 (11.65)		19 (8.89)		.02	
	Unemployed by choice	808 (9.66)		28 (13.08)		.02	
	Unemployed	845 (10.10)		32 (14.95)		.02	
**Chronic medical conditions, n (%)**							
	0	6261 (75.82)		110 (73.83)		.68	
	1	1738 (21.05)		32 (21.48)		.68	
	2	235 (2.85)		6 (4.03)		.68	
	3	24 (0.01)		1 (0.01)		.68	
Presence of chronic pain^c^, n (%)			851 (10.30)		28 (13.08)		.19
Panic attacks, n (%)			5712 (69.16)		147 (69.01)		.96
History of illicit drug use, n (%)			650 (7.87)		15 (7.04)		.80
History of suicide attempts, n (%)			195 (2.36)		8 (3.72)		.18

^a^Suicidal ideation was measured using item 9 of the Patient Health Questionnaire-9.

^b^Chronic pain status was missing in 1.28% (107/8366) of the patients in the treatment group.

^c^Participants (65/215, 30.2%) of the control group had missing data on racial minorities, gender, and education.

### Procedure

During a patient’s first session, a licensed professional prescribed psychiatric medications for the patient in the treatment group. Enrolled Brightside patients completed an initial digital intake that included clinically validated measures of depression and anxiety, as well as questions about clinical presentation, medical history, and demographics. Brightside’s proprietary precision-prescribing platform analyzes these data points using an empirically derived algorithm to provide real-time care guidelines and clinical decision support to providers via the digital platform. Brightside platform provides this decision support via a computerized symptom cluster analysis at treatment intake. On the basis of the analysis of presenting symptom clusters, as well as decision support based on the empirical literature, treatment recommendations are provided [45]. The algorithm uses a symptom cluster approach that places patients into a clinical subtype based on reported symptoms and prior medical trial results, along with comorbid medical conditions. Treatment recommendations were made according to the clinical subtype and weighted according to importance and severity and symptoms. Over the course of treatment, patients communicated with mental health providers both asynchronously via messaging and synchronously via video or telehealth sessions. The providers were psychiatrists, psychiatric nurse practitioners, or primary care physicians. These providers had an average of 12.8 contacts with patients in the first 12 weeks of treatment, which included check-in and case reviews, messaging, and video visits. Approximately half of the patients at Brightside were enrolled in a psychotherapy program. Brightside took a measurement-based approach to track long-term outcomes by prompting patients to complete periodic assessments during treatment. As such, providers were alerted in real time when patients reported improvement, failed to improve, worsened in their condition, or experienced high-risk symptoms such as SI. Most commonly prescribed regimens were monotherapy with escitalopram (1390/8366, 16.62%), bupropion (903/8366, 10.79%), sertraline (854/8366, 10.21%), or fluoxetine (502/8366, 6%), but many patients were started on some combination of different classes, with 425 different unique beginning treatment regimens and different doses. Follow-up surveys were collected at week 12 or any time before week 16.

### Measures

The Patient Health Questionnaire (PHQ-9) is a 9-item self-report measure used to assess the severity of depressive symptoms present within the prior 2 weeks, as outlined by the *Diagnostic and Statistical Manual of Mental Disorders, Fifth Edition* criteria. The PHQ-9 is a tool that is easy to use, but its validity remains widely confirmed [46]. Respondents rate items on a 4-point Likert scale (0-3), and total scores range from 0 to 27, with >9 indicating mild to low symptoms and >10 indicating moderate to severe symptoms [47]. The PHQ-9 shows strong validity, demonstrating 88% sensitivity and 88% specificity for major depressive disorder [47]. There is also evidence that the PHQ-9 can be used as a measure of antidepressant response [48]. In this study, patient responses to item 9 of the PHQ-9, “How often have you been bothered by thoughts that you would be better off dead or of hurting yourself in some way?” were used as a measure of SI severity [9,49]. Item 9 of the PHQ‐9 has been shown to predict suicide attempts and suicide-related deaths in an outpatient medical population [13]. For some analyses, a PHQ-8 score was calculated to avoid criterion contamination, which included the first 8 items and excluded the SI item.

The Generalized Anxiety Disorder-7 (GAD-7) scale is a 7-item self-report instrument used to assess the severity of anxiety symptoms present within the prior 2 weeks as outlined by *Diagnostic and Statistical Manual of Mental Disorders, Fifth Edition* criteria. Respondents rate items on a 4-point Likert scale (0-3), and total scores range from 1 to 21, with indications of 1 to 4 for minimal, 5 to 9 for mild, 10 to 14 for moderate, and 15 to 21 for severe symptoms [50]. The GAD-7 has shown strong reliability and validity, demonstrating 89% sensitivity and 82% specificity for GAD [51].

Basic demographic variables such as age, education, sex, and employment status were collected at baseline. In addition, individuals were asked if they had the following chronic health conditions: asthma, cancer, Crohn disease, irritable bowel syndrome, heart condition, obesity, or diabetes. A simple count variable was created with the number of chronic medical conditions endorsed. In addition, endorsement of either chronic pain or fibromyalgia was considered a variable representing chronic pain. Respondents were asked whether they used illicit substances and whether they currently experienced panic attacks. Finally, the patients were asked if they had ever attempted suicide in the past. Some participants (65/215, 30.2%) in the control group were missing the information about race, ethnicity, sex, and education level, whereas 1.28% (107/8366) of the participants in the treatment group were missing chronic pain status.

### Data Analyses

Data analyses were performed using SPSS (version 28; IBM Corp) to assemble the patient data sample, apply inclusion and exclusion criteria, and establish baseline versus follow-up survey outcomes. Brightside maintains deidentified databases for analytics that facilitate granular insights into clinical decisions, interactions, and outcomes. Assumptions for conducting regression models were assessed using visual inspection of distributions, a scatter plot of the residuals, and variance inflation factor values, as well as by examining potential multicollinearity among predictors. For the logistic regression models, the Box-Tidwell test was used to test whether the logit transform was a linear function of the predictor. In univariate general linear modeling of baseline SI severity, checking the assumptions revealed heteroscedasticity. As such, models were run using Box-Cox transformations [52]. The omnibus statistics were presented using these transformations. In all cases, the pattern and results were the same; as such, the means presented used nontransformed values for ease of interpretation.

First, we examined zero-order correlations between item 9, the SI item, on the PHQ-9 and change over time, as well as with various demographic, clinical, and medical variables. We also examined the correlations between item 9 and the other PHQ items. Pearson or point biserial correlation coefficients were calculated based on the variables included. Univariate general linear modeling was used to explore the independent effects of demographic and clinical variables on the baseline SI severity.

We then examined the relative rates of SI at baseline and 12 weeks, as well as the percentage change over time. Chi-square analyses compared relative proportions between groups. An analysis of covariance examined the treatment effect by group, on changes in SI over time, controlling for baseline age and PHQ-8 scores. Bonferroni corrections were used in all follow-up 2-tailed *t* tests. Mixed model analyses, controlling for age and PHQ-8 scores, were used to investigate the differences in SI over time by treatment group, education level, and employment status. These variables were chosen because they significantly differed between groups at baseline.

Next, using only those who endorsed SI at baseline (a score of ≥1), we calculated the proportion of people who *remitted* at 12 weeks (ie, they no longer endorsed any SI) and then examined potential predictors of remission. Chi-square analyses compared relative proportions among groups. Using the control group as the reference group, logistic regression with 95% CIs was used to examine bivariate relationships between SI remission and all previously described demographic, clinical, and medical variables. We then implemented a forward stepwise regression procedure to identify the independent predictors of remission during the study period, considering all the variables that were significant in the bivariate models.

### Ethics Approval

The WCG Institutional Review Board, Ethics Committee Panel 1, approved the retrospective research analysis of clinical data obtained by Brightside as part of routine clinical care (#1308524). The data were drawn from a deidentified clinical database.

## Results

### Predictors of SI Severity at Baseline

Correlations between the SI item on the PHQ-9 and all other items were examined (Table 2). Greater feelings of hopelessness, anhedonia, and feeling bad about oneself were most significantly correlated (*r*=0.24-0.37) with SI at baseline. Sleep issues and feeling tired or having low energy, although significant, had lower correlations with SI (*r*=0.13-0.14). In terms of demographic variables, advancing age and education were associated with less SI at baseline (*r*=−0.16) and 12 weeks (*r*=−0.10), but less improvement over time (*r*=−0.12 and −0.11, respectively). Although correlations between minority status (*r*=0.07) and employment status (*r*=0.05) and SI were significant, their magnitudes were small. Both the PHQ-8 and, to a lesser extent, the GAD-7 were significantly associated with SI (much more so at baseline, *r*=0.38 and 0.17, respectively) and with change over time (*r*=0.31 and 0.14, respectively). The number of chronic medical conditions was not significantly associated with SI or change over time. Endorsing either chronic pain or fibromyalgia was significantly associated with SI at baseline and week 12 but did not change over time, although the correlations were quite small (*r*=0.03-0.04). Having recent panic attacks and histories of illicit drug use or suicide attempt were significantly associated with SI at both time points, as well as change over time, although only correlations with baseline SI were of any magnitude (*r*=0.08-0.10).

A univariate general linear modeling was used to explore the independent effects of these demographic and clinical variables on baseline SI severity (Table 3). As GAD was highly correlated with PHQ-8 (*r*=0.35; *P*<.001), it was not included in the model. Similarly, having a comorbid panic disorder was correlated with sex (*r*=0.11; *P*<.001) and age (*r*=0.16; *P*<.001), so it was also excluded. The overall model was significant (*F*_12,7733_=132.11; *P*<.001) and accounted for 17% of the variance in the baseline SI. As shown in Table 3, the baseline PHQ-8 score was the most prominent predictor, controlling for all other variables, and accounted for 12% of the variance in baseline SI. Other predictors, while statistically significant, did not account for much of the variance; both age and education accounted for 2% of the variance.

**Table 2:** Zero-order correlations between suicidal ideation (SI) and demographic, medical, and clinical variables (N=8581).

			SI at baseline		SI at 12 weeks		Change in SI^a^
**Demographic data**							
	Age	−0.16^b^		−0.10^b^		−0.12^b^	
	Sex (female)	−0.00		–0.03^c^		0.01	
	Racial minority	0.07^b^		−0.03^c^		0.04^b^	
	Education (beyond high school)	−0.16^b^		−0.10^b^		−0.11^b^	
	Employment (less than full-time)	0.05^b^		0.05^b^		0.03^b^	
**PHQ^d^ items**							
	Anhedonia	0.24^a^		0.12^a^		0.18^a^	
	Feeling down, depressed, or hopeless	0.37^a^		0.15^a^		0.30^a^	
	Sleep issues	0.13^a^		0.07^a^		0.10^a^	
	Tired or low energy	0.14^a^		0.06^a^		0.11^a^	
	Appetite issues	0.18^a^		0.09^a^		0.14^a^	
	Feeling bad about self	0.36^a^		0.14^a^		0.30^a^	
	Trouble concentrating	0.18^a^		0.08^a^		0.15^a^	
	Psychomotor retardation or restless	0.23^a^		0.09^a^		0.19^a^	
**Baseline clinical factors**							
	PHQ-8	0.38^a^		0.14^a^		0.31^a^	
	Generalized Anxiety Disorder-7	0.17^a^		0.06^a^		0.14^a^	
**Medical factors**							
	Number of chronic medical conditions	0.01		0.02		–0.01	
	Chronic pain or fibromyalgia	0.03^b^		0.04^a^		0.01	
	Panic attacks	0.08^a^		0.06^a^		0.07^a^	
	History of illicit drug use	0.08^a^		0.04^a^		0.05^a^	
	History of suicide attempts	0.10^a^		0.04^a^		0.04^a^	

^a^Changes in SI represent changes from baseline to 12 weeks, with higher numbers representing decreased symptom severity over time on the PHQ-9 SI item.

^b^*P*<.001.

^c^*P*<.05.

^d^PHQ: Patient Health Questionnaire. PHQ-9 items are not the exact wording of the item. SI was assessed by responses to item 9 of the PHQ-9.

**Table 3:** Independent effects of demographic and clinical characteristics on baseline suicidal ideation (N=8581)^a^.

Factor	Sum squares	*F* (*df*)	*P* value	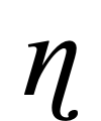 ^2^
Age	38.06	107.54 (1,7733)	<.001	0.02
Sex	0.21	0.60 (1,7733)	.44	0.00
Racial minority	2.44	6.90 (1,7733)	.01	0.00
Education	18.14	51.24 (1,7733)	<.001	0.02
Employment	0.56	1.51 (1,7733)	.22	0.00
Baseline PHQ-8^b^	373.39	1054.98 (1,7733)	<.001	0.12
Chronic pain	0.59	1.68 (1,7733)	.20	0.00
Number of medical conditions	1.06	1.00 (1,7733)	.39	0.00
Illicit drug use	8.12	22.94 (1,7733)	<.001	0.00
Suicide attempt	5.60	15.82 (1,7733)	<.001	0.00

^a^The dependent measure, baseline SI score, was transformed using the Box-Cox correction.

^b^PHQ-8: Patient Health Questionnaire-8.

### Treatment Effects on SI Severity

At baseline, 46.5% (100/215) of the participants in the control group and 47.12% (3942/8366) of the participants in the treatment group expressed SI (*χ*^2^_1_=0.0; *P*=.89). At 12 weeks, 34.4% (74/215) of the participants in the control group and 12.32% (1031/8366) of the participants in the treatment group expressed SI (χ^2^_1_=91.2; *P*<.001). Similarly, the percent change in SI scores was greater in the treatment group (*F*_1,8460_=43.60; *P*<.001; mean 37.54%, SD 52.55%) than that in the control group (mean 13.84%, SD 66.05%); 41.73% (3491/8366) of the participants in the treatment group had lessening of SI over time, compared with 27% (58/215) of the participants in the control group. Among the control group, 13.5% (29/215) of the participants expressed more severe SI at 12 weeks than at baseline, whereas only 2.53% (212/8366) of the participants in the treatment group did (χ^2^_1_=106.6; *P*<.001).

**Figure 1:**
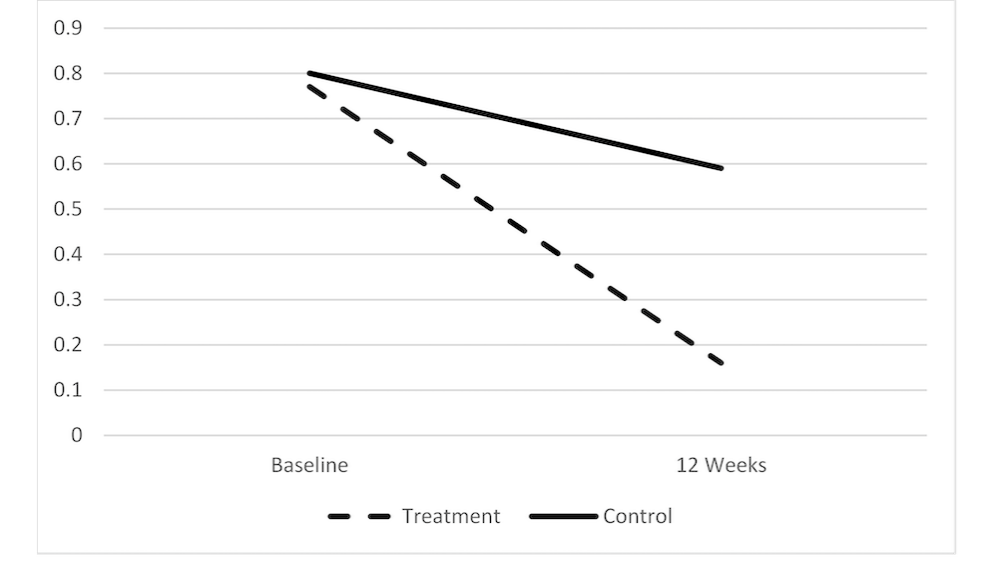
Average suicidal ideation scores over time by group.

In terms of emergence of SI in those who did not initially endorse it (n=4539), the control group had 15.6% (18/115) emergence, whereas the treatment group had only 2.98% (132/4424; χ^2^_1_=56.2; *P*<.001). Analysis of covariance, controlling for age and PHQ-8 scores, demonstrated a significant effect for the treatment group (F*_1,7809_*=145.46; *P*<.001) over time. Figure 1 illustrates the change in SI over time by group, with the treatment group clearly showing significantly greater improvement (ie, reduction of SI) over time.

**Figure 2:**
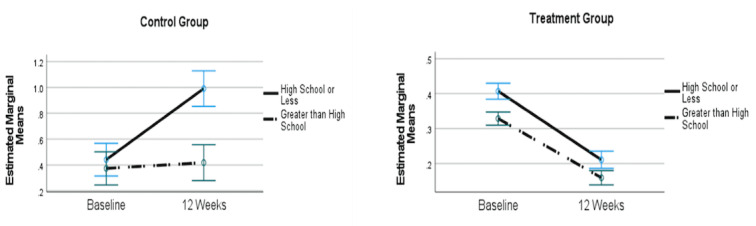
Interaction among group, time, and education level. Covariates appearing in the model were evaluated at age=32.03 years and PHQ-8 score=16.87. Error bars represent 95% CIs.

Mixed model analyses, controlling for age and PHQ-8, investigating differences in SI over time by treatment group, education level, and employment status revealed significant effects for the treatment group (F*_1,7740_*=46.85; *P*<.001), education level (F*_1,7740_*=19.97; *P*<.001), and employment status (F*_1,7740_*=6.96; *P*=.01). There were significant 2-way interactions, but these were further refined by a 3-way interaction among time, group, and education level (F*_1,7740_*=24.92; *P*<.001). Figure 2 shows that in the treatment group, those with both higher and lower levels of education reported less SI over time, but in the control group, education level interacted with time such that those with a high school education or less reported more SI over time (ie, got worse), whereas those with an above high school education level did not (and remained the same over time).

### Remission of SI

Among those who endorsed SI at baseline but then endorsed none at 12 weeks, 76.37% (3087/4042) of the total sample manifested this complete remission. Complete remission was observed in 77.19% (3043/3942) of the treatment group and 44% (44/100) of the control group (χ^2^_1_=59.5; *P*<.001).

On the basis of bivariate relationships, the following factors were significantly associated with SI remission: being in the treatment group, older age, being female, being White (as opposed to a racial minority), obtaining education beyond high school, and lower depression severity (as measured by PHQ-8 scores) at baseline (Table 4). Those in the treatment group were 4.3 times more likely to have SI remission than those in the control group (odds ratio [OR] 4.31, 95% CI 2.88-6.44). Women were approximately 1.4 times more likely to remit than men (OR 1.38, 95% CI 1.18-1.62). The variables that were significant in the bivariate models were entered into a binary logistic regression predicting remission using forward stepwise progression to determine the best predictors of remission, collectively. This model was 77% accurate in its classification. Table 5 shows that the treatment group, age, sex, and PHQ-8 scores were retained in the final model.

**Table 4:** Factors predicting suicidal ideation (SI) remission.

Predictor				No SI remission (n=955)		SI remission (n=3087)		Odds ratio (95% CI)	
**Treatment, n (%)**									4.31 (2.88-6.44)^a^
	Treatment group (n=3942)		899 (22.81)		3043 (77.19)				
	Control group (Ref^b^) (n=100)		56 (56)		44 (44)				
**Demographics**									
	Age (years), mean (SD)		29.43 (8.20)		30.81 (8.28)		1.02 (1.01-1.03)^a^		
	**Sex, n (%)**							1.38 (1.18-1.62)^a^	
		Female (n=2875)	626 (21.77)		2249 (78.23)				
		Male (n=1144)	318 (27.79)		826 (72.2)				
	**Minority status, n (%)**							0.79 (0.67-0.94)^a^	
		Minority (n=934)	250 (26.77)		684 (73.23)				
		White (Ref) (n=3079)	690 (22.41)		2389 (77.59)				
	**Education, n (%)**							1.37 (1.18-1.59)^a^	
		Beyond high school (n=2466)	523 (21.21)		1943 (78.79)				
		High school or less (Ref) (n=1547)	417 (26.96)		1130 (73.04)				
	**Employment, n (%)**							0.95 (0.76-1.19)	
		Unemployed (n=466)	114 (24.46)		352 (75.54)				
		Employed (Ref) (n=3575)	840 (23.5)		2735 (76.5)				
**Clinical, mean (SD)**									
	PHQ-8^c^		21.01 (3.98)		19.81 (4.10)		0.94 (0.92-0.96)^a^		
	GAD-7^d^		15.57 (4.42)		15.28 (4.46)		0.99 (0.97-1.00)		
**Medical condition, n (%)**									
	**Chronic pain or fibromyalgia**							0.81 (0.65-1.02)	
		Chronic pain (n=456)	123 (26.97)		333 (73.03)				
		No chronic pain (Ref) (n=3537)	818 (23.13)		2719 (76.87)				
	**Number of chronic medical issues**							0.91 (0.79-1.05)	
		0 (Ref) (n=2993)	695 (23.22)		2298 (76.78)				
		1 (n=853)	193 (22.63)		660 (77.37)				
		2 (n=106)	34 (32.08)		72 (67.92)				
		3 (n=12)	5 (41.67)		7 (58.33)				
	**Panic attacks**							0.79 (0.67-0.94)	
		Yes (n=2939)	724 (24.63)		2215 (75.37)				
		No (Ref) (n=1053)	217 (20.61)		836 (79.39)				
	**Illicit drug use**							0.86 (0.68-1.08)	
		Yes (n=405)	106 (26.17)		299 (73.83)				
		No (Ref) (n=3587)	835 (23.28)		2752 (76.72)				
	**Suicide attempts**							0.85 (0.57-1.27)	
		Yes (n=128)	34 (26.56)		94 (73.44)				
		No (Ref) (n=3867)	908 (23.48)		2959 (76.52)				

^a^Values indicate that the predictor significantly predicts SI remission at the 95% CI.

^b^Ref. represents the reference group.

^c^PHQ-8: Patient Health Questionnaire-8.

^d^GAD-7: Generalized Anxiety Disorder-7.

**Table 5:** Multivariate model predicting suicidal ideation remission.

Factor	Odds ratio^a^ (95% CI)
Treatment group	5.10 (3.03-8.43)
Age	1.02 (1.01-1.03)
Sex	1.49 (1.26-1.77)
PHQ-8^b^	0.94 (0.92-0.96)

^a^All odds ratios are *P*<.001; Nagelkerke *R*^2^=0.04.

^b^PHQ-8: Patient Health Questionnaire-8.

## Discussion

The objective of this study was to examine the impact of psychiatric care on SI, change in SI over time, and remission, as well as to investigate the relationship between various demographic and medical factors on SI and SI remission.

### SI Severity

Greater feelings of hopelessness, anhedonia, and feeling bad about oneself were most significantly correlated with SI at baseline. Sleep issues and feeling tired or having low energy, although significant, had lower correlations with SI. These patterns of associations between the SI item and other items of the PHQ-9 mirror those found in other studies with primary care patients who had depression or chronic pain and had completed the PHQ-9 [53], with the exception of anhedonia, which tended to have lower associations with SI in primary care patients [53]. Hopelessness has consistently been found to be a predictor of SI [54,55], although associations with actual suicide or attempts are mixed [56-58].

Associations between greater SI severity and younger age are consistent with national survey data finding that younger adults more frequently endorse SI than older adults [59]. As might be expected, educational levels beyond high school were associated with lower SI. The findings that advanced education and age were associated with less positive change in SI are difficult to reconcile though this may be because of range restriction (ie, there is less room to “improve”). In the Sequenced Treatment Alternatives to Relieve Depression (STAR*D) data, greater educational level and older age predicted improvement or lowering of SI [16].

Not surprisingly, both the PHQ-8 and, to a lesser extent, the GAD-7 were significantly associated with SI (much more so at baseline) and with change over time. The baseline PHQ-8 score was the most prominent independent predictor of SI, accounting for 12% of the variance. This reflects a consistent finding in the literature that depression severity is highly associated with SI [60] and with suicide-related outcomes [61] and illustrates the importance of antidepressant treatment.

The number of chronic medical conditions was not significantly associated with SI or change over time. Endorsing either chronic pain or fibromyalgia was significantly associated with SI at baseline and week 12 but did not change over time, although the overall correlations were quite small. This is counter to prior research [62,63] and may reflect the limited range (the maximum number of endorsed medical conditions was 3) or failure to consider the severity or burden of the medical conditions. Having recent panic attacks and histories of illicit drug use or suicide attempt were significantly associated with SI at both time points, as well as change over time, although only correlations with baseline SI were of any magnitude (*r*=0.08-0.10). Again, these findings are in line with prior research [22,64-66], although it is important to note that approximately 11% of those who attempt suicide deny ever having experienced any SI [66]. Therefore, SI is insufficient to explain all suicide attempts and completions.

### Treatment Effects

Although the groups were similar at baseline in terms of the presence of SI (47%) at baseline, after 12 weeks of treatment, only 12.32% (1031/8366) of the participants in the treatment group expressed any SI, compared with 34% (74/215) of the participants in the control group. These numbers are similar to a much smaller study investigating psychotherapy for depression [67], although that study’s participants had fewer people (30%) with SI at baseline than this study and less severe depression. In this study, 41.73% (3491/8366) of the participants in the treatment group showed improvement in SI severity over time, compared with 27% (58/215) of the participants in the control group. The baseline rate of SI and the percentage of people who were improved at the last visit were very similar to those in the STAR*D trial [68], which was conducted in person, as compared with this study conducted virtually. In terms of the emergence of new SI in those not initially endorsing it, 15.6% (18/115) of the participants in the control group showed emergent SI, compared with only 2.98% (132/4424) of the participants in the treatment group. These proportions are similar to the 1.3% emergence seen in the STAR*D trial at 12 weeks [67,68], although the STAR*D trial was treated in a more homogenous fashion in person. Further research is needed to replicate these promising treatment effects with virtual treatment and clinical decision support tools for more tailored precision prescribing.

Although the treatment group improved over time regardless of various demographic variables, in the control group, after controlling for age and depression severity, those with less education worsened over time. Greater education levels are protective against many adverse outcomes, including SI [69-71]. Telehealth interventions, such as the Brightside platform used with the treatment group in this study, may be indicated for those at risk because of lower education.

### Remission

Those in the treatment group were 4.3 times more likely to remit than those in the control group (OR 4.31, 95% CI 2.88-6.44). Zisook et al [40] found similar rates of remission in those receiving 12 weeks of escitalopram plus placebo, bupropion sustained release plus escitalopram or venlafaxine extended release plus mirtazapine. Collectively, these findings suggest strong evidence that antidepressant medications have a positive impact on SI. Whether selective serotonin reuptake inhibitors and other new-generation antidepressants alter the risk of suicide in adults is certainly controversial [38,39,72,73], with the most recent review suggesting that they are associated with a higher risk of suicide [17]. These authors contend that publication bias and conflicts of interest likely contribute to systematic underestimation of risk. Although this study involved SI and did not evaluate suicide attempts or completion to the extent that SI is predictive of suicide attempts and actual suicide [9,10], these data are more optimistic.

Treatment was clearly the biggest predictor of SI remission. Other factors found to be significantly associated with SI remission were older age, being female, being White, obtaining education beyond high school, and having lower depression severity at baseline. Women and those with advanced education beyond high school were about 1.4 times (OR 1.38, 95% CI 1.18-1.62) more likely to remit than men and those without advanced education (OR 1.37, 95% CI 1.18-1.59). In the STAR*D trial, remission of depressive symptoms was more likely in those who were White, female, employed, or had higher levels of education or income [74]. Although not specifically focused on SI, these factors are similar to those found in this study.

### Limitations

The primary limitation of this study is that there was no random assignment to treatment, and alternative explanations for any observed treatment effects are possible. Although comparison to a control group that completed assessments on the same schedule as the treatment group, and which was largely equivalent to the treatment group at baseline, reduced the likelihood that any effects were because of engagement on line or other Hawthorne effects, the control group did not engage with providers. As the participants were not randomized, there is potential for confounding. For example, participants in the control group were more likely to be unemployed and have no education beyond high school. As it is unclear why the control group participants did not pursue treatment, one possibility is that they had less scheduling flexibility or less ability to take time off to attend treatment.

Another limitation is the inability to directly compare different medications, as this was a clinical sample with >400 different combinations of medications. Although this prevents generalizability to specific medication groups, it does speak to the ability of antidepressant treatment, as rendered in this novel virtual treatment regimen, to positively affect SI. An additional limitation, however, is that patients may have had other treatments such as psychotherapy that were not assessed. In addition, a specific measure of SI, such as the Beck Scale for Suicide Ideation or the Scale for Suicide Ideation [75], would have improved the study rather than a single item from a global measure of depression. Further research will be required to study the impact of this prescription model on suicide attempt.

### Treatment Implications and Conclusions

Depression severity is the primary driver of SI, relative to demographic and other clinical or medical factors. Certainly, many clinicians may be reluctant to prescribe antidepressants in those with SI because of the perceived risk of working with patients who are suicidal. These results address, at least to some degree, these concerns. The results of this study, as well as those of others, are consistent with the efficacy of psychiatric care administered via a telehealth platform with decision support. In antidepressant trials, depression severity mediates the effect of antidepressant medication on suicide risk [76,77], so treating the depressive symptoms is paramount. In this study and others, antidepressant medication positively affected SI severity in the vast majority of people. Finally, additional efforts should be made to treat those with lower levels of education, as they are more likely to have increasing SI over time without such treatment. These findings highlight the importance of considering covariates, or subpopulations, when considering SI [78].

Finally, these results align with a growing body of literature demonstrating the effectiveness of using a telehealth platform for providing mental health services [79-82]. Clinical decision support tools have demonstrated efficacy in depression outcomes in primary care and general practices [83-85]. The strengths of this study include its size, use of a control group, and the novel use of a telehealth platform. Further research and refinement, ideally via randomized controlled trials, are needed.

## Abbreviations

GAD: general anxiety disorder
GAD-7: Generalized Anxiety Disorder-7
OR: odds ratio
PHQ: Patient Health Questionnaire
SI: suicidal ideation
STAR*D: Sequenced Treatment Alternatives to Relieve Depression
